# Evaluating the functional, sexual and seasonal variation in the chemical constituents from feces of adult Iberian wolves (*Canis lupus signatus*)

**DOI:** 10.1038/s41598-023-33883-9

**Published:** 2023-04-24

**Authors:** Isabel Barja, Ana Piñeiro, Aritz Ruiz-González, Amaia Caro, Pilar López, José Martín

**Affiliations:** 1 Unidad de Zoología, Departamento de Biología, Universidad Autónoma de Madrid, Madrid, Spain; 2 Research Centre in Biodiversity and Global Change (CIBC-UAM), Universidad Autónoma de Madrid, Madrid, Spain; 3 Escuela de Medicina Veterinaria, Facultad de Ciencias de la Vida, Universidad Andrés Bello, Santiago de Chile, Chile; 4 Department of Zoology and Animal Cell Biology, University of the Basque Country (UPV/EHU), C/ Paseo de La Universidad 7, 01006 Vitoria-Gasteiz, Spain; 5 Departamento de Ecología Evolutiva, Museo Nacional de Ciencias Naturales, CSIC, Madrid, Spain

**Keywords:** Ecology, Behavioural ecology

## Abstract

Chemical signals deposited in feces play an important role in intraspecific and interspecific communication of many mammals. We collected fresh feces of adult wolves from wild breeding groups. All samples visually identified as belonging to wolves were subsequently identified to species level by sequencing a small fragment of mtDNA and sexed typing DBX6 and DBY7 sex markers. Using gas chromatography-mass spectrometry (GC–MS), we identified 56 lipophilic compounds in the feces, mainly heterocyclic aromatic organic compounds, such as indole or phenol, but also steroids, such as cholesterol, carboxylic acids and their esters between n-C_4_ and n-C_18_, aldehydes, alcohols and significant quantities of squalene and α-tocopherol, which would increase the chemical stability of feces on humid substrates. There was variability in the number and proportions of compounds between sexes, which could be indicative of their function as chemical signals. We also found variability in different reproductive states, especially in odorous compounds, steroids and α-tocopherol. Feces with a presumed marking function had higher proportions of α-tocopherol and steroids than feces with non-marking function. These compounds could be involved in intragroup and intergroup communication of wolves and their levels in feces could be directly related with the wolf’s sex and physiological and reproductive status.

## Introduction

Chemical signals play a very important role in intraspecific and interspecific communication of many animal species^1–5^. In many mammals, different semiochemicals are incorporated into feces, urine or other scent marks, so that they remain on the substrate with the aim of scent-marking the boundaries of their territory or attracting potential mates^6,7^. For a carnivore mammal, scent marking its environment is very important for several reasons; to delimit territories, thus maintaining the separation between individuals and groups^8^, to reaffirm the possession of resources^9,10^, and to signal their social status^11,12^ or a certain physiological, reproductive or emotional state^13,14^. In addition, these odorous signals may help individuals to orient themselves within their own territories or simply make them feel safe therein^15^. In many carnivores, there is a close association between dominance and scent marking, and in some species, such as the wolf (*Canis lupus*), only high-ranking individuals (i.e., the alpha pair), show marking behavior with urine^16,17^ and feces^12^, through which they continuously signal their social status to the rest of the group members^18^.

Chemical communication plays an important role in the social organization and spatial distribution of wolves^12,15,19–21^. Indeed, they largely depend on smell, which allows them to acquire information about their environment and communicate within the group and with other groups^22,23^. Wolves invest their time and energy in an odorous marking strategy that guarantees the maximum probability of detection of fecal signals^19,24^. They deposit feces and urine on visually conspicuous and elevated substrates, which improve their function as marks^15–17,20,24,25^. For example, in the wild, Iberian wolves select plants with greater diameter and height as a substrate to deposit feces, increasing the effectiveness of feces as visual signals, and facilitating the dispersion of the smell by the wind and the increase of the surface of evaporation^24^. In addition, feces are accumulated in strategic sites, such as crossroads where the probability of being detected by conspecifics is greater^19^.

The analyses of fecal volatile compounds have reaffirmed the function of feces as source of chemical signals, due to the large number of chemical compounds found in them. These include a high proportion of aromatic organic heterocyclic compounds (i.e., with benzene rings), aldehydes, low-weight fatty acids and alcohols, which are strongly odoriferous compounds^26^, which are similar to those found in feces of domestic dogs^27^. Many of these volatile compounds originate in the secretions of the anal sacs and are later incorporated into the stool during defecation^18^, and these chemical compounds have also been identified in the secretions of the anal sacs of wolves^28,29^. However, there are many other highly volatile compounds identified in the secretions of the anal sacs of the wolf but not found in the feces, probably because they evaporated quickly from the feces due to their exposure to environmental conditions^26^. Also, secretions of the anal sacs can be deposited independently to the defecation and are not always deposited in all the excrements (only in less than 10% of the feces)^30,31^. Thus, the functions of the fecal marks and the anal sacs secretions may be independent, or be used in different contexts, which suggest a double role in chemical communication. The compounds in secretions of the anal sacs deposited independently of the feces are more volatile and could act as a short-term warning signal intended directly for nearby individuals, while the more persistent compounds found in feces seem to be used in long-term territorial marking^30^.

Adult male wolves, especially alpha males, deposit anal secretions more frequently when defecating than females or juveniles^30^. Moreover, feces of young wolves and adults are clearly different, with adults having more volatile aromatic compounds and fatty acids that are absent in feces of the offspring^26^. Thus, the volatile compounds present in the feces seem to function as very important chemical signal in the intraspecific communication of the wolf, giving information about sex, age, endocrine status and their individual identity^28^.

In this study, we aimed to examine several aspects of the function of fecal scent marks as chemical signals in the Iberian wolf (*Canis lupus signatus*). The compounds present in wolf fecal samples collected in the wild were analyzed using gas chromatography-mass spectrometry (GC–MS) to answer the following questions:

1. Are there intersexual differences in the presence and abundance of volatile compounds in the feces of adult wolves?
2. Do the presence and abundance of these compounds in the feces vary depending on the reproductive status of the individuals?
3. Are the feces with a presumed marking function (i.e., deposited on conspicuous substrates, above ground level, at crossroads or as re-marking) the only ones that play a role in the chemical communication of the wolves?

## Results

### Species and sexual identification

Out of the 73 fecal samples analyzed by molecular methods, 67 samples were successfully sexed (42 males and 25 females). In addition, after sequencing a 440 bp mtDNA fragment from fecal DNA, we effectively identified 56 samples as wolf. All the sequences obtained in this study matched with sequences published in previous studies^32–37^ and are available from the Dryad repository, as well as their correspondence with the published sequences (see Data availability section). Thus, unequivocal species identification was possible in 76.7% of the samples. Moreover, although 17 (23.3%) feces could not be genetically identified, in no case did the genetic analyses indicate that any of these fecal samples came from another of the species of carnivores found in the study area such as fox, wildcat or European marten. Also, all fecal samples were collected in the vicinity of the *rendezvous sites* and their morphology matched that of wolf feces. Thus, we considered that all the 94 samples collected very likely belonged to wolves and all of them were used for the chemical analyses.

### Chemical compounds in feces of adult wolves

We found a total of 56 lipophilic compounds in 94 fresh feces considered to belong to adult Iberian wolves (Table 1). The main compounds were 11 aromatic heterocyclic compounds (37.6% of the TIC), 24 carboxylic acids and their esters between n-C_4_ and n-C_20_ (22.3%) and eight steroids (21.7%). In addition, we also found five aldehydes (6.3%), squalene (6.0%), α-tocopherol (3.7%) and other minor compounds, such as two alcohols (0.8%), two amides (0.8%), cyclic octaatomic sulfur (0.7%) and one ketone (0.02%) (Table 1). On average, the five most abundant compounds were indole (28.5%), cholesterol (11.5%), squalene (6.0%), hexadecanoic acid (5.9%) and phenol (5.0%).

**Table 1:** Relative proportion (mean ± SE) of lipophilic compounds found in hexane extracts of feces of all adult Iberian wolves (*Canis lupus signatus*), and of males and females separately in fecal samples from which sex was determined.

RT (min)	Compound	MW (g/mol)	All samples (n = 94)	Males (n = 42)	Females (n = 25)
Aromatic heterocyclic compounds					
16.0	Benzaldehyde*	106.12	0.03 ± 0.02	0.08 ± 0.07	0.04 ± 0.03
19.5	Phenol*	94.11	4.98 ± 0.66	5.06 ± 1.29	5.66 ± 1.39
24.1	4-Methyl phenol (= p-cresol)*	108.14	0.68 ± 0.23	1.48 ± 0.76	0.65 ± 0.30
28.7	2-Piperidinone	99.13	0.53 ± 0.15	0.91 ± 0.40	1.05 ± 0.58
29.2	Quinoline*	129.16	2.32 ± 0.47	2.83 ± 0.77	3.33 ± 1.31
31.2	Indole*	117.15	28.47 ± 2.27	28.12 ± 4.13	36.51 ± 6.51
31.9	1,2,3,4-Tetrahydro-quinoline	133.19	0.10 ± 0.06	0.09 ± 0.06	–
32.2	Benzenepropanoic acid, ethyl ester*	178.23	0.14 ± 0.08	0.48 ± 0.29	0.10 ± 0.05
33.7	Benzenepropanoic acid*	150.18	0.18 ± 0.07	0.43 ± 0.22	0.38 ± 0.23
36.5	1,3-Dihydro-2H-indol-2-one	133.15	0.11 ± 0.05	0.20 ± 0.12	0.36 ± 0.20
41.8	2,4-Dihydroxy-3,6-dimethyl-benzoic acid, methyl ester	182.17	0.09 ± 0.04	0.05 ± 0.03	0.08 ± 0.05
Carboxylic acids and their esters					
5.3	Butanoic acid, ethyl ester*	116.16	0.20 ± 0.04	0.14 ± 0.06	0.19 ± 0.10
6.3	Butanoic acid*	88.11	1.10 ± 0.31	1.28 ± 0.49	1.60 ± 0.73
8.9	3-Methylbutanoic acid*	102.13	0.43 ± 1.12	0.74 ± 0.37	0.92 ± 0.33
9.8	2-Methylbutanoic acid*	102.13	0.33 ± 0.10	0.67 ± 0.29	0.55 ± 0.27
10.0	Pentanoic acid, ethyl ester*	130.18	0.06 ± 0.04	0.17 ± 0.17	0.01 ± 0.01
13.1	Pentanoic acid*	102.13	0.10 ± 0.05	–	0.43 ± 0.28
38.2	Dodecanoic acid*	200.31	0.08 ± 0.03	0.12 ± 0.11	0.16 ± 0.08
42.4	Tetradecanoic acid*	228.37	1.02 ± 0.31	1.73 ± 1.03	1.41 ± 0.63
43.9	Pentadecanoic acid, ethyl ester*	270.45	0.01 ± 0.01	–	–
44.4	Pentadecanoic acid*	242.4	0.03 ± 0.01	0.04 ± 0.03	–
44.7	9-Hexedecenoic acid, methyl ester*	268.43	0.01 ± 0.01	–	–
45.1	Hexadecanoic acid, methyl ester*	270.45	0.02 ± 0.01	0.03 ± 0.02	–
45.9	9-Hexadecenoic acid, ethyl ester*	284.48	0.61 ± 0.14	0.43 ± 0.21	0.40 ± 0.29
46.0	9-Hexadecenoic acid*	254.41	1.55 ± 0.58	0.71 ± 0.27	0.21 ± 0.15
46.3	Hexadecanoic acid, ethyl ester*	284.48	0.64 ± 0.25	0.14 ± 0.09	0.09 ± 0.06
46.5	Hexadecanoic acid*	256.42	5.92 ± 1.23	6.50 ± 2.76	4.90 ± 2.86
48.3	9-Octadecenoic acid, methyl ester*	296.49	0.57 ± 0.24	0.53 ± 0.49	–
48.8	Octadecanoic acid, methyl ester*	294.47	0.02 ± 0.01	0.02 ± 0.02	–
49.4	9,12-Octadecadienoic acid, ethyl ester*	308.50	0.95 ± 0.30	0.70 ± 0.24	0.45 ± 0.30
49.5	9-Octadecenoic acid, ethyl ester*	310.51	3.51 ± 0.85	3.03 ± 1.31	0.56 ± 0.25
49.9	Octadecanoic acid, ethyl ester*	312.53	0.16 ± 0.05	0.11 ± 0.07	0.01 ± 0.01
49.9	9-Octadecenoic acid*	282.46	4.79 ± 1.37	2.45 ± 2.39	1.35 ± 1.24
52.1	5,8,11,14-Eicosatetraenoic acid, ethyl ester*	332.52	0.13 ± 0.06	0.03 ± 0.03	0.08 ± 0.08
52.6	3-Hydroxy-octadecanoic acid, methyl ester	286.40	0.06 ± 0.03	0.08 ± 0.08	0.01 ± 0.01
Steroids					
60.4	Cholesta-4,6-dien-3-ol*	384.63	0.89 ± 0.61	0.55 ± 0.21	0.30 ± 0.12
60.7	Cholesta-3,5-diene*	368.64	1.06 ± 0.17	1.43 ± 0.31	2.31 ± 0.64
63.3	Cholestan-3β-ol*	388.67	0.26 ± 0.13	0.59 ± 0.45	0.05 ± 0.05
64.0	Cholesterol*	386.65	11.47 ± 1.22	12.92 ± 1.99	6.81 ± 2.04
64.6	Cholestan-3-one*	386.65	1.42 ± 0.36	2.11 ± 1.13	1.05 ± 0.65
66.1	Cholest-4-en-3-one*	384.64	4.03 ± 0.56	5.67 ± 1.48	3.32 ± 1.09
66.7	Cholesta-4,6-dien-3-one*	382.62	0.64 ± 0.17	0.43 ± 0.21	0.15 ± 0.06
68.8	Unidentified steroid (189, 203, 218, 313, 409, 424)	??	1.94 ± 0.38	0.89 ± 0.34	1.61 ± 1.16
Aldehydes					
5.4	Hexanal*	100.16	0.08 ± 0.03	0.17 ± 0.02	0.01 ± 0.01
38.6	Tetradecanal*	128.21	0.05 ± 0.02	0.03 ± 0.02	0.10 ± 0.07
40.9	Pentadecanal*	226.40	0.15 ± 0.05	0.03 ± 0.02	0.26 ± 0.15
42.9	Hexadecanal*	240.42	3.38 ± 0.49	2.84 ± 0.97	2.95 ± 1.31
46.9	Octadecanal*	268.48	2.65 ± 0.32	2.48 ± 0.59	3.31 ± 1.16
Terpenoids					
59.2	Squalene*	410.72	6.00 ± 1.12	3.19 ± 0.59	8.03 ± 3.57
Tocopherols					
63.7	α-Tocopherol*	430.71	3.75 ± 0.47	4.05 ± 0.99	5.22 ± 1.55
Alcohols					
42.3	Tetradecanol*	214.39	0.06 ± 0.04	0.10 ± 0.08	0.03 ± 0.02
44.3	Hexadecanol*	242.44	0.73 ± 0.20	1.21 ± 0.58	1.63 ± 0.53
Amides					
50.3	9-Octadecenamide*	281.48	0.01 ± 0.01	0.02 ± 0.02	0.04 ± 0.02
53.1	Nonadecanamide	297.52	0.81 ± 0.25	1.19 ± 0.58	0.04 ± 0.02
48.2	Cyclic octaatomic sulfur	256.52	0.67 ± 0.20	0.64 ± 0.39	1.22 ± 0.47
Ketones					
40.5	2-Pentadecanone	226.40	0.02 ± 0.01	0.04 ± 0.03	0.05 ± 0.02

The relative amount of each component was determined as the percent of the total ion current (TIC). An asterisk after the compound name indicates that the identification was confirmed with standards. The other compounds were tentatively identified based on mass spectra and retention times (RT). Characteristic ions (*m/z*) are reported for an unidentified steroid. *MW* molecular weight.

The number of compounds identified in a single fecal sample ranged between 2 and 43 (mean ± SE = 16 ± 1 compounds/fecal sample). All major compounds (> 5%) were detected in most samples, although the presence and relative proportions of some chemicals show a high inter-sample variability.

The PCA analysis of the transformed areas of all the compounds, extracted 6 principal components (PCs) with eigenvalues greater than two, which together accounted for 47.6% of the variance (Table 2). The correlations of the relative proportions of the volatile compounds with the PCs are shown in Table 2.

**Table 2:** Principal components analysis (PCA) for relative proportions of compounds found in feces of Iberian wolves (*Canis lupus signatus*). Correlations between variables (compounds) and the principal components significant at P < 0.00001 are marked in bold.

Compound	PC-1	PC-2	PC-3	PC-4	PC-5	PC-6
Benzaldehyde	0.39	0.01	0.28	0.04	**0.71**	− 0.13
Phenol	0.23	**− 0.52**	− 0.13	0.19	0.03	− 0.04
4-Methylphenol (= p-cresol)	**0.61**	− 0.15	− 0.08	− 0.01	0.08	0.15
2-Piperidinone	**0.59**	0.01	**0.49**	0.05	0.25	0.12
Quinoline	**0.47**	− 0.39	0.02	− 0.02	0.01	0.04
Indole	0.07	− 0.27	**− 0.75**	− 0.08	− 0.20	0.02
1,2,3,4-Tetrahydro-quinoline	**0.58**	− 0.03	0.01	0.11	− 0.11	− 0.35
Benzenepropanoic acid, ethyl ester	**0.65**	− 0.03	− 0.12	0.07	0.14	0.10
Benzenepropanoic acid	**0.70**	0.07	0.09	0.08	0.27	0.26
1,3-Dihydro- 2H-indole-2-one	**0.58**	− 0.05	0.06	0.01	**0.49**	0.32
2,4-Dihydroxy-3,6-dimethyl-benzoic ac. methyl ester	**0.58**	− 0.14	− 0.10	0.08	0.34	− 0.27
Butanoic acid, ethyl ester	0.28	0.32	− 0.01	− 0.02	0.01	0.01
Butanoic acid	**0.75**	0.10	0.43	0.02	0.01	0.09
3-Methyl-butanoic acid	**0.82**	− 0.04	0.06	0.08	0.15	0.11
2-Methyl-butanoic acid	**0.67**	0.04	0.06	0.08	0.15	0.11
Pentanoic acid, ethyl ester	0.05	**0.47**	0.18	− 0.14	− 0.06	− 0.08
Pentanoic acid	0.30	− 0.03	0.09	− 0.12	0.16	0.36
Dodecanoic acid	0.32	0.03	**0.54**	0.01	**0.61**	− 0.09
Tetradecanoic acid	0.19	0.07	**0.72**	0.07	0.27	0.24
Pentadecanoic acid, ethyl ester	− 0.01	0.23	0.06	− 0.10	− 0.23	0.02
Pentadecanoic acid	− 0.08	− 0.08	**0.72**	0.03	0.01	− 0.03
9-Hexadecenoic acid, methyl ester	− 0.01	0.10	0.20	0.06	0.04	− 0.23
Hexadecanoic acid, methyl ester	0.03	0.36	0.15	0.08	0.22	− 0.24
9-Hexadecenoic acid, ethyl ester	− 0.14	**0.56**	0.18	0.20	0.22	− 0.04
9-Hexadecenoic acid	0.34	− 0.02	**0.53**	0.17	− 0.06	0.02
Hexadecanoic acid, ethyl ester	0.16	0.42	− 0.17	0.19	0.04	0.17
Hexadecanoic acid	0.03	0.11	**0.73**	0.01	0.25	0.15
9-Octadecenoic acid, methyl ester	− 0.15	**0.64**	0.18	0.16	− 0.04	− 0.02
Octadecanoic acid, methyl ester	0.01	**0.62**	− 0.05	− 0.03	− 0.03	− 0.04
9,12-Octadecadienoic acid, ethyl ester	− 0.07	**0.46**	0.23	0.22	0.21	0.04
9-Octadecenoic acid, ethyl ester	0.09	**0.59**	0.13	0.19	0.11	0.18
Octadecanoic acid, ethyl ester	0.05	**0.66**	− 0.03	− 0.13	0.01	− 0.06
9-Octadecenoic acid	0.11	0.19	**0.61**	0.06	− 0.09	0.38
5,8,11,14-Eicosatetraenoic acid, ethyl ester	− 0.14	0.01	0.20	0.02	0.08	0.13
3-Hydroxy-octadecanoic acid, methyl ester	0.17	− 0.11	0.17	0.08	0.02	**0.74**
Cholesta-4,6-dien-3-ol	0.27	0.09	− 0.07	0.26	0.23	0.15
Cholesta-3,5-diene	0.27	− 0.16	− 0.05	**0.56**	0.15	0.21
Cholestan-3β-ol	− 0.07	0.04	− 0.13	0.14	0.20	− 0.03
Cholesterol	0.10	0.02	0.07	**0.74**	− 0.08	− 0.04
Cholestan-3-one	− 0.27	0.12	0.08	0.38	0.28	0.12
Cholest-4-en-3-one	0.02	0.14	0.11	**0.62**	0.06	0.03
Cholesta-4,6-dien-3-one	− 0.13	0.19	0.39	0.42	0.27	0.23
Unidentif. steroid (189, 203, 218, 313, 409, 424)	− 0.04	0.08	0.01	− 0.01	0.26	− 0.05
Hexanal	0.02	− 0.04	**0.49**	− 0.05	0.08	− 0.18
Tetradecanal	0.36	0.06	0.33	− 0.01	0.16	**0.68**
Pentadecanal	0.41	− 0.01	0.21	0.05	**0.68**	0.10
Hexadecanal	0.01	0.14	0.07	0.29	− 0.08	0.09
Octadecanal	0.01	0.03	− 0.05	0.28	− 0.23	0.10
Squalene	0.02	0.06	− 0.07	0.17	− 0.04	0.01
α-Tocopherol	0.25	− 0.13	0.07	**− 0.53**	0.06	− 0.06
Tetradecanol	0.42	− 0.04	− 0.14	0.11	0.35	**0.53**
Hexadecanol	0.38	0.01	− 0.11	0.02	0.47	0.21
Octadecenamide	0.24	0.07	0.19	− 0.01	**0.73**	0.09
Nonadecanamide	− 0.04	0.27	0.13	**0.46**	− 0.05	− 0.02
Cyclic octaatomic sulfur	0.37	0.25	− 0.01	0.01	0.31	0.12
2-Pentadecanone	0.08	0.04	0.14	0.11	**0.59**	0.41
Eigenvalues	10.39	5.42	3.47	2.78	2.36	2.21
Explained variance (%)	18.55	9.69	6.24	4.97	4.21	3.94

### Chemical compounds in feces of males and females

Males and females had similar lipophilic compounds in their feces, although four minor compounds were only found in one of the sexes. The main classes of compounds found in feces of males and females were also similar, but the relative proportions of these compounds were slightly different (Table 1). The main classes of compounds found in feces of males were heterocyclic aromatic organic compounds (39.7%), steroids (24.6%) and carboxylic acids and their esters (19.6%), whereas in females the main classes found were heterocyclic aromatic organic compounds (48.2%), steroids (15.6%) and carboxylic acids and their esters (13.3%). The five most abundant compounds in feces of males were indole (28.1%), cholesterol (12.9%), hexadecanoic acid (6.5%), cholest-4-en-3-one (5.7%) and phenol (5.1%), whereas the main compounds in feces of females were indole (36.5%), squalene (8.0%), cholesterol (6.8%), phenol (5.1%), and α-tocopherol (5.2%) (Table 1).

**Figure 1:**
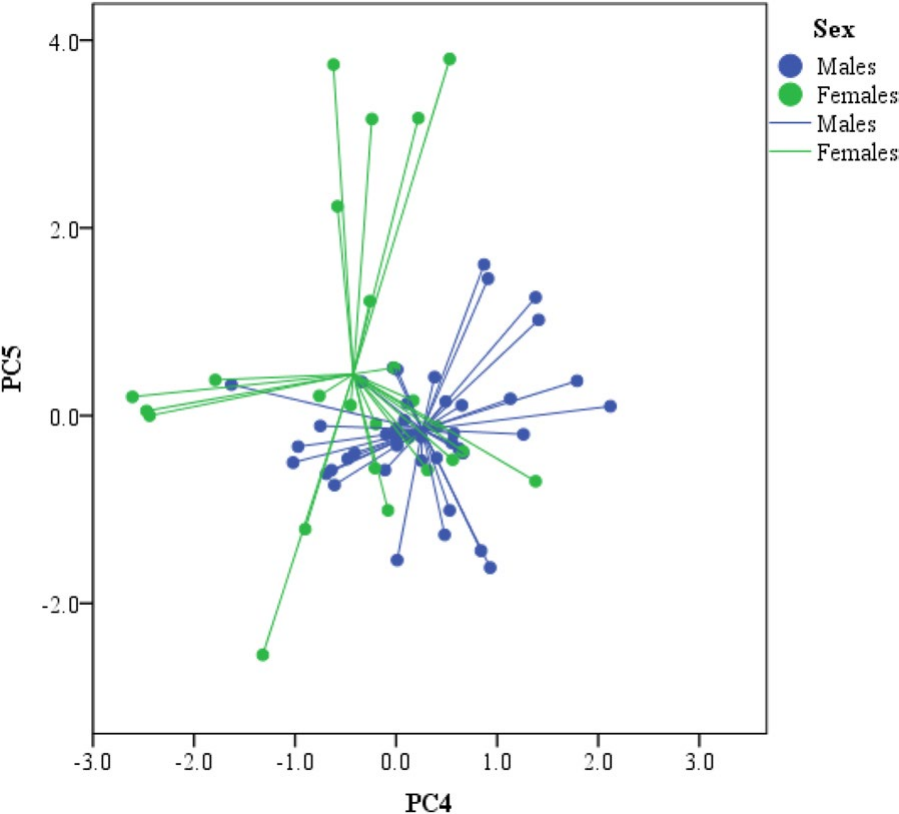
PC4 and PC5 individual factor scores extracted from the PCA for relative proportions of lipophilic compounds in feces of male (blue) and female (green) Iberian wolves (*Canis lupus signatus*). Individual points are related to the centroid for all samples of each sex.

The PERMANOVA analysis based on the resemblance matrix comparing samples of each sex showed significant differences in the overall proportion of compounds in the entire chemical profile between males and females (pseudo F_1,65_ = 5.69, P = 0.02). Also, results from the PCA showed that there were significant intersexual differences in the compounds described by PC4 (ANOVA, F_1,65_ = 11.14, P = 0.014; Fig. 1), but not in the other PCs (ANOVA, at least P > 0.15 in all cases). Thus, according to the significant correlations of the compounds with the PCs, males had higher levels of steroids such as cholesterol and cholesta-4-en-3-one than females (Table 1). Moreover, a discriminant analysis based on these compounds described by PC4 alone assigned correctly the sex of 79% of feces of males and 31% of feces of females (Wilks' λ = 0.85, F_1,65_ = 11.30, P = 0.013).

### Seasonal differences

The PERMANOVA analysis based on the resemblance matrix comparing samples of each season showed that there were significant differences in the overall proportion of compounds among the three seasons (pseudo F_2,91_ = 1.58, P = 0.037). However, pairwise permutational post-hoc tests showed that there were significant differences between the reproductive and the non-reproductive season (P = 0.01) and between the reproductive and the breeding seasons (P = 0.039), but there were not significant differences between the breeding and the non-reproductive seasons (P = 0.51). The CAP analysis assigned 49% of the chemical profiles into the correct season using Euclidean distances between samples (permutational test, δ_1_^2^ = 0.49, P = 0.043, using leave-one-out cross-validation and m = 30 axis).

A further two-way PERMANOVA restricted to samples that could be sexed (n = 67) confirmed that there were significant overall seasonal differences and significant differences between sexes independently of the seasonal variation (season: pseudo F_2,61_ = 2.15, P = 0.002; sex: pseudo F_1,61_ = 1.89, P = 0.027; season × sex: pseudo F_2,61_ = 0.78, P = 0.79).

**Figure 2:**
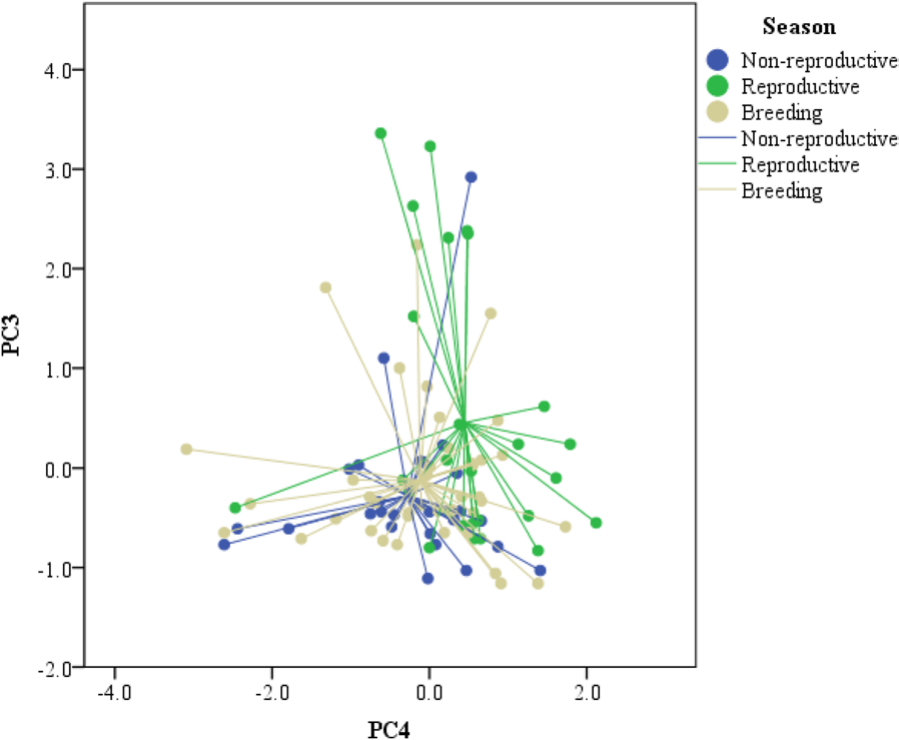
PC4 and PC3 individual factor scores extracted from the PCA made for relative proportions of lipophilic compounds in feces of adult Iberian wolves (*Canis lupus signatus*) depending on the reproductive status of individuals: non-reproductive (blue), reproductive (green) or breeding (grey). Individual points are related to the centroid for all samples of each reproductive state.

The analysis of seasonal variation in the PCs resulting from the PCA of compounds showed that there were significant seasonal differences in the compounds described by PC4 (ANOVA, F_2,91_ = 4.28, P = 0.017) and by PC3 (ANOVA, F_2,91_ = 4.45, P = 0.014) (Fig. 2), but not in the other PCs (ANOVA, P > 0.25 in all cases). Thus, during the reproductive season there were significant lower relative proportions of indole, but significant higher relative proportions of hexanal and several fatty acids (PC3) and of cholesterol and α-tocopherol (PC-4) than during the non-reproductive (Tukey's tests, P = 0.02 for both PCs) and the breeding seasons (P < 0.05 for both PCs) (Table 3). These two seasons did not differ significantly between them (P > 0.78 for both PCs). A discriminant analysis based on compounds described by PC3 and PC4 alone classified samples into the correct season for 44% of samples of the reproductive period and 92% of samples of the non-reproductive and breeding seasons (Wilks' λ = 0.82, F_4,180_ = 4.54, P = 0.0016).

**Table 3:** Relative proportions (mean ± SE) of compounds in feces of Iberian wolves (*Canis lupus signatus*) depending on the reproductive status of the individuals.

Compound	Breeding (n = 41)	Non-reproductive (n = 26)	Reproductive (n = 27)
Benzaldehyde	0.06 ± 0.04	0.00 ± 0.00	0.01 ± 0.01
Phenol	5.14 ± 0.89	6.92 ± 1.69	2.47 ± 0.83
4-Methylphenol (= p-cresol)	0.28 ± 0.14	1.59 ± 0.76	0.22 ± 0.17
2-Piperidinone	0.66 ± 0.28	0.42 ± 0.30	0.48 ± 0.20
Quinoline	3.23 ± 0.91	1.77 ± 0.48	1.49 ± 0.84
Indole	28.83 ± 3.62	36.66 ± 4.61	19.74 ± 3.53
1,2,3,4-Tetrahydro-quinoline	0.21 ± 0.15	0.02 ± 0.02	0.01 ± 0.01
Benzenepropanoic acid, ethyl ester	0.05 ± 0.03	0.21 ± 0.20	0.01 ± 0.01
Benzenepropanoic acid	0.17 ± 0.10	0.09 ± 0.07	0.29 ± 0.19
1,3-Dihydro- 2H-indole-2-one	0.11 ± 0.08	0.03 ± 0.03	0.07 ± 0.05
2,4-Dihydroxy-3,6-dimethyl-benzoic acid methyl ester	0.19 ± 0.09	0.02 ± 0.02	–
Butanoic acid, ethyl ester	0.15 ± 0.05	0.22 ± 0.08	0.24 ± 0.08
Butanoic acid	1.20 ± 0.46	1.22 ± 0.82	0.67 ± 0.33
3-Methyl-butanoic acid	0.55 ± 0.22	0.18 ± 0.10	0.24 ± 0.14
2-Methyl-butanoic acid	0.35 ± 0.14	0.26 ± 0.13	0.42 ± 0.24
Pentanoic acid, ethyl ester	0.03 ± 0.02	0.00 ± 0.00	0.17 ± 0.16
Pentanoic acid	0.07 ± 0.06	0.14 ± 0.14	–
Dodecanoic acid	0.07 ± 0.04	–	0.20 ± 0.11
Tetradecanoic acid	0.66 ± 0.29	0.83 ± 0.41	1.89 ± 0.92
Pentadecanoic acid, ethyl ester	0.02 ± 0.01	–	0.01 ± 0.01
Pentadecanoic acid	0.02 ± 0.02	0.03 ± 0.03	0.05 ± 0.02
9-Hexadecenoic acid, methyl ester	0.01 ± 0.01	–	0.02 ± 0.01
Hexadecanoic acid, methyl ester	0.02 ± 0.01	–	0.03 ± 0.02
9-Hexadecenoic acid, ethyl ester	0.53 ± 0.18	0.31 ± 0.19	1.09 ± 0.39
9-Hexadecenoic acid	0.37 ± 0.21	0.20 ± 0.11	4.81 ± 1.93
Hexadecanoic acid, ethyl ester	1.11 ± 0.57	0.23 ± 0.14	0.39 ± 0.15
Hexadecanoic acid	4.52 ± 1.66	5.87 ± 2.20	8.80 ± 2.90
9-Octadecenoic acid, methyl ester	0.20 ± 0.09	1.02 ± 0.59	0.76 ± 0.61
Octadecanoic acid, methyl ester	–	0.05 ± 0.05	0.02 ± 0.02
9,12-Octadecadienoic acid, ethyl ester	0.58 ± 0.20	0.76 ± 0.39	1.79 ± 0.98
9-Octadecenoic acid, ethyl ester	5.01 ± 1.78	1.10 ± 0.53	3.91 ± 1.20
Octadecanoic acid, ethyl ester	0.17 ± 0.07	0.13 ± 0.06	0.19 ± 0.12
9-Octadecenoic acid	5.26 ± 2.33	3.67 ± 2.36	5.70 ± 2.62
5,8,11,14-Eicosatetraenoic acid, ethyl ester	0.15 ± 0.07	–	0.23 ± 0.18
3-Hydroxy-octadecanoic acid, methyl ester	0.04 ± 0.04	–	0.15 ± 0.10
Cholesta-4,6-dien-3-ol	1.74 ± 1.43	0.28 ± 0.13	0.26 ± 0.15
Cholesta-3,5-diene	0.92 ± 0.23	1.25 ± 0.39	0.88 ± 0.26
Cholestan-3β-ol	0.49 ± 0.28	0.03 ± 0.03	0.18 ± 0.18
Cholesterol	11.16 ± 1.64	10.57 ± 1.93	13.47 ± 3.07
Cholestan-3-one	1.45 ± 0.72	0.77 ± 0.35	2.09 ± 0.61
Cholest-4-en-3-one	3.71 ± 0.93	4.64 ± 0.98	4.35 ± 1.11
Cholesta-4,6-dien-3-one	0.43 ± 0.18	0.28 ± 0.14	1.37 ± 0.52
Unidentified steroid (189, 203, 218, 313, 409, 424)	3.44 ± 0.77	0.81 ± 0.37	0.98 ± 0.35
Hexanal	0.04 ± 0.02	0.01 ± 0.01	0.15 ± 0.09
Tetradecanal	0.06 ± 0.04	0.01 ± 0.01	0.06 ± 0.04
Pentadecanal	0.22 ± 0.10	–	0.20 ± 0.10
Hexadecanal	3.25 ± 0.64	2.83 ± 0.84	4.48 ± 1.21
Octadecanal	2.64 ± 0.49	2.44 ± 0.58	3.20 ± 0.68
Squalene	5.88 ± 1.70	6.15 ± 2.43	3.46 ± 0.57
α-Tocopherol	2.51 ± 0.54	3.91 ± 0.84	5.40 ± 1.19
Tetradecanol	0.09 ± 0.08	0.07 ± 0.07	0.03 ± 0.02
Hexadecanol	0.73 ± 0.30	0.74 ± 0.50	0.48 ± 0.27
9-Octadecenamide	0.01 ± 0.01	–	0.03 ± 0.02
Nonadecanamide	0.37 ± 0.19	1.00 ± 0.58	1.40 ± 0.61
Cyclic octaatomic sulfur	0.83 ± 0.39	0.25 ± 0.15	0.91 ± 0.39
2-Pentadecanone	0.03 ± 0.02	–	0.02 ± 0.01

### Marking function of feces

**Figure 3:**
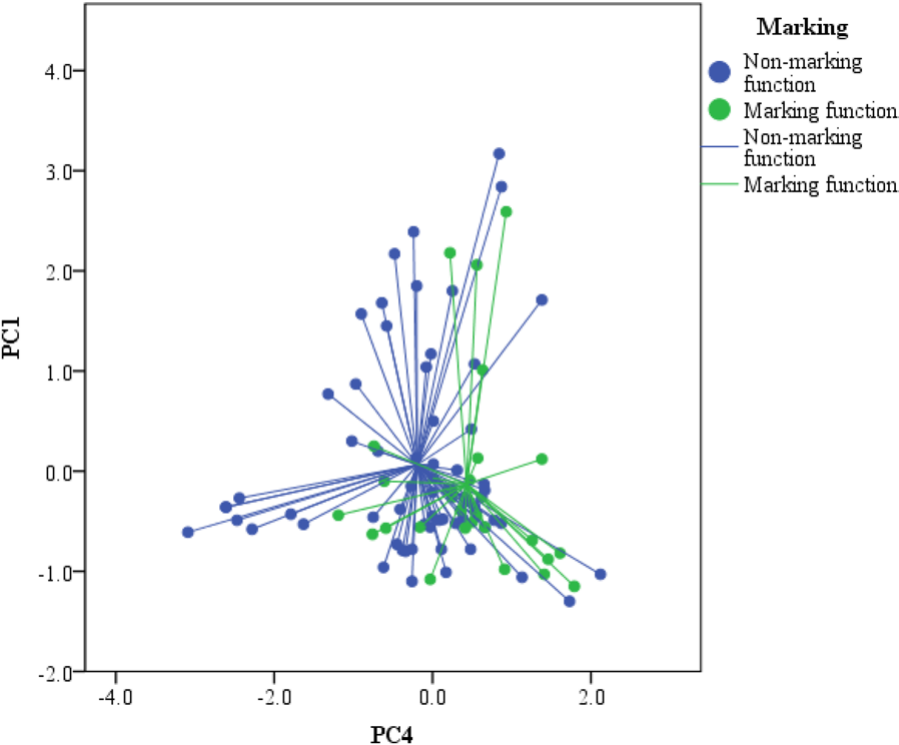
PC4 and PC1 individual scores extracted from the PCA made for relative proportions of lipophilic compounds in feces of adult Iberian wolves (*Canis lupus signatus*) depending on the presumably marking function (non-marking: blue; marking: green) of these feces in intraspecific communication. Individual points are related to the centroid for all samples of each marking function.

The PERMANOVA analysis based on the resemblance matrix comparing samples of feces deposited with a presumably marking function (i.e., feces left on conspicuous substrates, above ground level, at crossroads and/or remarking other feces) with those of feces with a non-marking function (i.e., feces that were on inconspicuous substrates and/or at ground level, off crossroads, non-remarking) showed that there were not significant differences in the overall proportion of compounds between feces with different functions (pseudo F_1,90_ = 0.74, P = 0.79). Nevertheless, the PCA showed significant differences in the compounds described by the PC4 (ANOVA, F_1,90_ = 8.03, P = 0.006; Fig. 3), but not in the other PCs (P > 0.12 for all), suggesting that feces with a presumably marking function had higher proportions of α-tocopherol and cholesterol than feces with a no-marking function (Table 4). A discriminant analysis based on these compounds described by PC4 alone classified correctly 95% of samples from feces without a marking function, but only 18% of feces with a marking function (Wilks' λ = 0.92, F_1,90_ = 8.03, P = 0.006).

**Table 4:** Relative proportions (mean ± SE) of compounds in feces of Iberian wolves (*Canis lupus signatus*) depending on the presumably marking function of these feces in intraspecific communication.

Compound	Non-marking function (n = 64)	Marking function (n = 28)
Benzaldehyde	0.03 ± 0.03	0.02 ± 0.02
Phenol	5.31 ± 0.86	3.96 ± 1.07
4-Methylphenol (= p-cresol)	0.80 ± 0.33	0.28 ± 0.16
2-Piperidinone	0.48 ± 0.17	0.72 ± 0.37
Quinoline	2.43 ± 0.61	2.10 ± 0.86
Indole	29.49 ± 2.93	27.48 ± 4.00
1,2,3,4-Tetrahydro-quinoline	0.14 ± 0.10	0.02 ± 0.02
Benzenepropanoic acid, ethyl ester	0.09 ± 0.08	0.08 ± 0.04
Benzenepropanoic acid	0.14 ± 0.07	0.29 ± 0.18
1,3-Dihydro- 2H-indole-2-one	0.04 ± 0.02	0.18 ± 0.12
2,4-Dihydroxy-3,6-dimethyl-benzoic acid methyl ester	0.08 ± 0.05	0.11 ± 0.06
Butanoic acid, ethyl ester	0.20 ± 0.05	0.19 ± 0.08
Butanoic acid	1.13 ± 0.42	0.94 ± 0.44
3-Methyl-butanoic acid	0.31 ± 0.13	0.49 ± 0.21
2-Methyl-butanoic acid	0.29 ± 0.10	0.48 ± 0.25
Pentanoic acid, ethyl ester	0.09 ± 0.07	0.01 ± 0.01
Pentanoic acid	0.10 ± 0.07	0.02 ± 0.02
Dodecanoic acid	0.05 ± 0.02	0.18 ± 0.11
Tetradecanoic acid	1.01 ± 0.28	1.20 ± 0.85
Pentadecanoic acid, ethyl ester	0.01 ± 0.01	0.02 ± 0.02
Pentadecanoic acid	0.03 ± 0.01	0.01 ± 0.01
9.Hexadecenoic acid, methyl ester	0.01 ± 0.01	–
Hexadecanoic acid, methyl ester	0.02 ± 0.01	0.01 ± 0.01
9-Hexadecenoic acid, ethyl ester	0.74 ± 0.19	0.42 ± 0.22
9-Hexadecenoic acid	2.13 ± 0.86	0.41 ± 0.19
Hexadecanoic acid, ethyl ester	0.76 ± 0.37	0.47 ± 0.18
Hexadecanoic acid	6.46 ± 1.55	4.68 ± 2.29
9-Octadecenoic acid, methyl ester	0.57 ± 0.32	0.64 ± 0.39
Octadecanoic acid, methyl ester	0.03 ± 0.02	–
9,12-Octadecadienoic acid, ethyl ester	1.17 ± 0.43	0.60 ± 0.35
9-Octadecenoic acid, ethyl ester	3.67 ± 1.12	3.30 ± 1.38
Octadecanoic acid, ethyl ester	0.14 ± 0.05	0.20 ± 0.11
9-Octadecenoic acid	5.33 ± 1.76	2.37 ± 1.64
5,8,11,14-Eicosatetraenoic acid, ethyl ester	0.17 ± 0.08	0.07 ± 0.07
3-Hydroxy-octadecanoic acid, methyl ester	0.06 ± 0.04	0.07 ± 0.07
Cholesta-4,6-dien-3-ol	1.15 ± 0.92	0.31 ± 0.15
Cholesta-3,5-diene	1.01 ± 0.21	1.03 ± 0.29
Cholestan-3β-ol	0.27 ± 0.17	0.29 ± 0.20
Cholesterol	9.27 ± 1.13	16.53 ± 3.07
Cholestan-3-one	1.68 ± 0.50	0.98 ± 0.51
Cholest-4-en-3-one	4.24 ± 0.71	4.17 ± 1.07
Cholesta-4,6-dien-3-one	0.72 ± 0.24	0.55 ± 0.25
Unidentified steroid (189, 203, 218, 313, 409, 424)	1.73 ± 0.47	2.62 ± 0.72
Hexanal	0.02 ± 0.01	0.15 ± 0.09
Tetradecanal	0.03 ± 0.03	0.08 ± 0.05
Pentadecanal	0.13 ± 0.06	0.21 ± 0.09
Hexadecanal	3.61 ± 0.61	3.34 ± 0.95
Octadecanal	2.60 ± 0.40	3.03 ± 0.60
Squalene	4.38 ± 0.97	7.49 ± 2.56
α-Tocopherol	3.61 ± 0.58	4.15 ± 0.95
Tetradecanol	0.08 ± 0.06	0.04 ± 0.02
Hexadecanol	0.75 ± 0.28	0.51 ± 0.22
9-Octadecenamide	–	0.05 ± 0.02
Nonadecanamide	0.78 ± 0.29	1.01 ± 0.54
Cyclic octaatomic sulfur	0.39 ± 0.14	1.43 ± 0.60
2-Pentadecanone	0.02 ± 0.01	0.02 ± 0.01

## Discussion

Our results show that the feces of Iberian wolf feces (*Canis lupus signatus*) contain a wide variety of chemical compounds, among which there was a large proportion of very odorous compounds such as aromatic heterocyclic organic compounds, aldehydes, low molecular weight fatty acids and alcohols^26^. Chemical signals that mediate social and reproductive behaviors are often species-specific mixtures or bouquets of structurally similar, but differentially expressed, compounds^7^. Our multivariate analyses suggested that subtle variations in this mixture of compounds in feces of wolves might allow them to identify, in many cases with high reliability, the sex and reproductive status of the individual that produced the signal. This reaffirms the important role that feces may play in the chemical communication of wolves, probably at least when feces were produced with an intended signaling function.

Some of the abundant highly odoriferous compounds found in the feces of wolves might not be directly produced by the anal glands, but rather would result from bacterial transformation of other compounds found in the anal sacs or coming from the prey remains in the intestine^26^. This could be the case of the aminoacids tryptophan and tyrosine transformed by bacteria to indole and phenol respectively, benzoic acid transformed to benzaldehyde, or some fatty acids produced by bacteria^38^. Nevertheless, these compounds secondarily produced by bacterial fermentation, and that disappear from secretions when an antibiotic treatment is provided^28^, may also have a signaling function in the short-term. These compounds likely are directly intended for individuals present at the moment of defecation, as it occurs with some compounds from the anal gland secretions of foxes^39^ and wolves^28^.

On the other hand, the high molecular weight compounds present in feces are more stable and, therefore, they could play an important role in the long-term chemical communication directed to other individuals that could find the feces in the future. Thus, the odor signal will remain longer than if it was restricted to the highly volatile low molecular weight compounds, such as those present in urine and secretions of the anal sacs^28,29,40^. Among these compounds found in feces, there was a significant proportion of squalene and α-tocopherol. Squalene is a well-known lipophilic fixative that could stabilize the other lipid fractions by decreasing oxidation and, therefore, increasing the chemical stability of feces on wet substrates, as it has been showed in scent marks of other animals^41,42^. Tocopherol is also an antioxidant^43^ that has also been found in the feces of other mammals, such as in goats, *Capra hircus*^44^ and in the anal glands of wolverines, *Gulo gulo*^45^.

In addition, the differences in the proportion of α-tocopherol in feces could be related to the diet, endocrine status or condition of the individual^43^. The dominant wolves are the ones that feed first on the captured prey and consume the best parts of the prey, being the subordinates forced to consume lower quality food, less rich in proteins, and in scarce circumstances to fast^46^. Also, dominant wolves are the only individuals that breed within each group and those that are responsible for marking the territory^12,47^. The α-tocopherol is a chemical compound of dietary origin, and studies carried out with reptiles showed how individuals with a higher quality diet secreted higher proportions of vitamin E in their chemical signals^48^. This suggests that the presence of this compound in the secretions involved in chemical communication is expensive and, therefore, may depend on the quality and health of the individuals. In some insects, the nutritional status of males affects the quality of their pheromones in the attraction of a couple^49,50^ and, similarly, in the mountain rock lizard (*Iberolacerta montícola*) the quality of the femoral secretions of males as a female attractor increases when supplementing their diet with vitamin D^51^.

Multivariate analyses showed that intersexual differences in the overall chemical profiles of feces, which are likely based on the significant differences found in the relative proportions of some shared compounds, could allow wolves to discriminate the sex of the signaling individual by chemosensory stimuli in many cases. Among the shared compounds, α-tocopherol was more abundant in feces of females, while some steroids, such as cholesterol and cholesta-4-en-3-one, were relatively more abundant in males. This suggests that the different proportions of these compounds in feces may be related to the levels of reproductive hormones, or to sex-specific differences in the metabolism of these compounds, similar to what occurs with the intersexual differences in the presence of glucocorticoids in feces^52^. These differences may allow wolves to signal their sex, but also their endocrine status and condition for marking territories or attracting potential mates.

Differences were also observed in the number and proportion of compounds present in the feces of wolves in their different reproductive stages. Multivariate analyses showed that feces produced in the reproductive season had different chemical profiles than those produced in the non-reproductive or breeding season, which did not differ. Some of the highly odoriferous compounds found in feces of wolves, such as, hexanal and several fatty acids, were more abundant during the reproductive season, when they might contribute to make the scent signal more conspicuous to conspecifics during this season. However, when the adult wolves were outside of the reproductive period, the number of volatile compounds in feces and the relative proportion of steroids and α-tocopherol decreased, while proportions of indole increased. Indole was present in all wolf feces, including those from pups^26^. These results could suggest that, only during the reproductive season, individuals would be interested in investing in signalling their physiological status using some expensive compounds that may play a major role in chemical communication and, therefore, they would decrease their allocation to feces outside of the reproductive period.

The results obtained also support our hypothesis that not all feces are deposited with a presumed marking function. We had previously classified some feces as olfactory-visual marks based on the physical characteristics of the substrate where feces were deposited and their distribution in the wolves' territory, which increase the visibility and detection by residents and intruders wolves^24,53^. Multivariate analyses showed that the overall chemical profile of these feces was similar to that of feces without a marking function. However, we found in feces with a presumably marking function a relative higher proportion of α-tocopherol, squalene and steroids, than in the rest of feces without an assigned marking function. Therefore, it seems that when feces were deposited with a signaling function, individuals would more often invest in secreting compounds that are more expensive but that may contribute to preserve the signal for longer and have a presumably function in chemical communication.

The relative proportion of α-tocopherol (= vitamin E) in feces differed between sexes, increased during the reproductive period, and was also higher in feces with a presumed marking function. All of these results suggest that α-tocopherol may play an important role in the intra-group and inter-group communication of the wolves and may have three main functions. First, α-tocopherol has very important functions in metabolism^43^, so allocating high levels of this compound in feces can be indicative of the quality of individuals (good nutrition, good health, etc.). Similarly, in ocellated lizards (*Timon lepidus*) the release of high levels of vitamin E in its signaling secretions is directly related to the quality of the immune system of males^26^. Second, levels of α-tocopherol may be related to reproduction and partner search, as suggested by the high levels of this compound during the reproductive season. Wolves could use this feature to assess not only the individual quality and reproductive status of the producer of the feces, but also to indirectly estimate the quality of a territory, that is, the quality of the available food^54^ and might show preference for areas marked with signals that contain high levels of vitamin E. Alternatively, α-tocopherol might not really be important as a true signaling compound in feces, but its antioxidant properties would increase the duration and intensity of the information provided by other compounds present in the secretions^41^. In any case, it is likely that only individuals with a good quality and health may afford to divert high concentrations of vitamin E from metabolism to deposit them in their feces and be able to maintain their territory. This could suggest that the secretion of this compound with feces entails a cost for individuals, conferring reliability on the olfactory marks, which would allow their evolution as reliable sexual signals.

In summary, the differences between sexes and between seasons in the chemical profiles of feces of wolves, which are probably related to their physiological and reproductive status, reaffirm the function of feces as olfactory marks in this species. The greater proportion of certain compounds in feces deposited with a presumably marking function, with respect to those that do not, reaffirm the theory that not all feces are deposited with a potential signaling function. The presence of high levels of α-tocopherol in feces deposited during the reproductive season and with a marking function, suggests its possible role as a sexual signal indicator of the individual “quality” of wolves or their territories, or as a preservative and amplifier of the signal of other compounds. However, future studies are required to reveal the role that vitamin E and other compounds may play in the communication of wolves. These should include examining the behavioral responses of wolves to experimental scent marks manipulated with different proportions, or mixes, of the different compounds with a potential signaling function. We also require studies that relate the natural or manipulated physiological state of a given individual with the compounds found in its feces.

## Material and methods

### Study area and collection of fecal samples

The study was carried out in two mountain areas in northwestern Spain; Natural Park Os Montes do Invernadeiro and its surroundings (Ourense prov.; 42° 07′ 52″ N/07° 19′ 09″ W; 880–1700 m.a.s.l.) and Sierra de la Culebra (Zamora prov.; 41° 53′ 54″ N/06° 20′ 01″ W; 800–1200 m.a.s.l). These areas have a high density of wild ungulates (red deer, roe deer, and wild boar) and carnivores (fox, wildcat and European marten), specially holding the highest density of wolves in the Iberian Peninsula and throughout Western Europe.

We collected fecal samples from five wolf-breeding groups; four in the Sierra de la Culebra and one in the Natural Park Os Montes do Invernadeiro. In each group, fresh feces of adult wolves were collected monthly, from May 2007 to December 2008, along forest tracks and firebreaks that were frequently transited by wolves. The fecal samples were collected without handling the animals. To discriminate which group the fecal samples belonged to, the pathways prospected to collect the feces were established in each group in the vicinity of the *rendezvous sites*, since the wolves defend territorial groups and there is no overlap between them^46,47^. The wolves (pups and adults) remain in the * rendezvous sites* from July to September and, sometimes, until mid-October. Therefore, in these zones there was a high level of activity of pups and adults, which facilitates the collection of very fresh feces (with mucosal cuticle, see below). Thus, almost all feces were collected during the summer period (in summer). Therefore, weather conditions cannot differentially affect to the freshness of the feces. During May and June of both years, we searched the *rendezvous sites*. These were located in the center of the territory of each group of wolves, where there was a great activity of pups and adults (footprints, excrements, bony remains of prey, trampling of vegetation, tracks, etc.), which facilitated the collection of very fresh feces and decreased the likelihood of confusion with the excrements of other carnivores. Nevertheless, to discriminate the feces of wolves from those of other species of sympatric carnivores, their size and shape were taken into account, not collecting samples from feces with a diameter of less than 2.5 cm and a length less than 25 cm. Moreover, despite all these precautions, the feces collected were analyzed by molecular techniques to identify the species and sex (see below).

The transects were inspected in an off-road vehicle twice a day (at dawn and at dusk), so the time from deposition to collection of feces was less than 12 h, given that the wolves show their peaks of greatest activity at dawn and dusk^47^. To avoid potential losses of compounds in old feces exposed to the environment for long periods of time^41^, during this study only very fresh feces were collected for the chemical analyses. To assess whether a fecal pellet was fresh, we examined several characteristics that all together made fresh feces unmistakable from older feces. Fresh feces were very moist and shiny, had a mucosal cuticle on the outside, and a very strong and characteristic smell. Also, fresh feces broke down quickly due to that they had organic matter as the predominant content. However, the main remains that remained in long-time exposed feces were indigestible prey parts (hair and hooves). The exposure to environmental conditions, such as sunlight and time faeces were exposed to the environment, made feces much clearer and lost its scent. On the other hand, when exposed to high temperatures, typical of summer, tended to lose its layer of mucus quickly (I. Barja, pers. obs.). Furthermore, feces were sometimes associated with urine and/or scratches on the ground, acting as composite signals to conspecifics^15,21^. This allowed us to determine the freshness of some feces, because urine quickly disappeared from the plants (substrates selected by wolves for marking)^24^ and the soil; and the scratches dried, becoming compact promptly. Whenever there was snow (November and December), it preserved the volatile compounds in fresh feces for a longer time, even those of low molecular weight. In addition, during the collection of fecal samples in the study area, we have set camera traps, for another purpose, nearby to the collection transects, which allowed us to confirm the freshness of the scats collected. Therefore, following all these premises, we could ensure that the collected feces were very fresh, which minimizes the potential loss of volatile compounds as exposure to environmental factors^41^.

We classified feces in two groups. (a) possible marking function in intraspecific visual and chemical communication, and (b) simple excretions. We considered that feces had a marking function when deposited on conspicuous substrates (plants, rocks, trunks, etc.), above ground level, at crossroads and/or over feces of conspecifics (over-marking). We considered that a substrate was conspicuous when it was the most outstanding of all the available ones found within a 2 m radius circle around the scat^12,15,20,24^. Therefore, we considered the rest of the substrates non-conspicuous. Additionally, we considered feces as marking cues if they occurred on a substrate > 4 cm above ground level^15,55^ and at a crossroad of two or more trails^20^. We considered that there was over-marking or re-marking when wolves defecated over one or several previous fecal marks^52^. We collected around 10 g of each fresh scat and stored it in a portable refrigerator, loaded with ice. Then, we kept the samples at – 20 °C refrigerator for further analyses.

All fecal samples collected were of unknown origin with respect to the individual that had produced. To minimize pseudoreplication and avoid bias in the study due to a small number of different prospective individuals, five wolf breeding groups whose group sizes ranged between 6 and 14 individuals (I. Barja, unpublished data) were followed. The group size was obtained by direct observation of the groups at dusk and dawn. Likewise, the alpha pair of each group is the only one that reproduces, and the rest of the members collaborate in the breeding, providing food for the pups and the female when they are in the den and in the *rendezvous sites* (cooperative breeding)^47,56^. Therefore, in the access lanes to these zones, we can often find several fresh excrements belonging to different individuals, thus ensuring that the collection of samples does not distort our results.

### Identification of the species and sex of the producers of the feces using molecular techniques

To reliably verify that the visually identified fecal samples correspond to wolves, avoiding confusion with feces of other sympatric carnivores, we conducted a species identification step consisting of sequencing mitochondrial DNA (mtDNA) control region on the fecal samples collected in the field. A subsample of each excrement was placed in tubes with 96% ethanol and stored at – 20 °C until processed. The extraction of DNA from fecal samples was carried out using an extraction kit based on silica membranes and adapted to non-invasive samples (QIAamp DNA Stool Mini Kit, Qiagen), following the manufacturer’s guidelines.

To determine the species origin of the fecal samples, a 440 bp fragment of the mitochondrial DNA control region was sequenced following the methodology described in Vilá et al.^32^. The experimental part consisted in the amplification of DNA using the PCR technique (Polymerase Chain Reaction) and the use of universal primers Thr-L 15926 and DL-H 16340^32^ and in its subsequent sequencing through the application of the commercial kit dRhodamine Terminator Cycle Sequencing Ready Reaction (Applied Biosystems), in an automatic sequencer ABI PRISM Model 3130 (Applied Biosystems). The success of the DNA amplification was verified by gel electrophoresis. The cleaning and purification of the amplified product was carried out according to the combined method of alkaline phosphatase and exonuclease I (ExoSAP-IT) developed by Amersham Biosciences, to eliminate the primers and the excess of deoxynucleotides that could interfere in the subsequent sequencing reaction. Species assignment was made thanks to the comparison of the sequences obtained with reference sequences of dogs and wolves obtained in previous studies^32–37^ and with those deposited in the GenBank databases for different mammalian species (http://www.ncbi.nlm.nih.gov/) and using the BLAST 2.0 program (http://www.ncbi.nlm.nih.gov/BLAST/).

To determine the sex of the samples identified as produced by wolves, we used the method described by Seddon^57^, designed specifically for sexual determination in fecal samples. To this end, two specific canine markers were amplified using the PCR technique: the DBX intron6 (249 bp), which identifies the X chromosome in males and females, and the DBY intron7 (118 bp) that identifies the Y chromosome in males. The success of the DNA amplification was verified by the electrophoretic migration of the amplified product in 1.5% agarose gels. We identified as males those samples that presented the bands corresponding to the X and Y chromosomes, and as females those samples that exclusively presented the band corresponding to the X chromosome. As there are several problems associated with the low quantity and quality of DNA extracted from scats, all samples were processed in duplicate. Samples whose identification by agarose gel was doubtful—due to the presence of faint or fuzzy bands—and all female samples—to confirm the real absence of the Y allele—were also genotyped with two replicates using an automatic sequencer (ABI PRISM 3130, Applied Biosystems). For the visualization and detection of the fragments corresponding to the X and Y chromosomes, the program GENEMAPPER version 4.0 (Applied Biosystems) was used.

### Chemical analyses of volatiles in feces

We transferred a small amount (approximately 2 g) of each fecal sample to a clean 2 ml chromatography glass vial to which 250 μl of *n*-hexane was added (Sigma, capillary GC grade). Each vial was closed with a Teflon-lined stopper before mixing the solution for 1 min using a vortex. Thereafter, the vial was placed in a fridge for 10 min to rest until the solid material that was not dissolved precipitated at the bottom of the vial. We extracted the supernatant clear liquid phase with a glass syringe and transferred it to a clean vial closed with a Teflon-lined stopper. We also made blank control vials using the same procedure, but without adding fecal material, to compare with the wolf samples. Thus, we were able to detect contaminants from the handling procedure or potential impurities in the solvent.

To analyze samples, we used a Finnigan-ThermoQuest Trace 2000 gas chromatograph (GC) fitted with a poly (5% diphenyl/95% dimethylsiloxane) column (Supelco, Equity-5, 30 m length, 0.25 mm ID, 0.25 mm film thickness) and a Finnigan-ThermoQuest Trace 2000 mass spectrometer (MS) as detector. We used helium at a constant flow rate of 0.8 ml/min as the carrier gas. We injected 2 µl of each sample in splitless mode with an inlet temperature of 250 °C. The oven of the GC was programmed so that the temperature was kept initially at 45 °C for 15 min, and then increased at a rate of 5 °C/min until a final temperature of 280 °C, which was kept for 15 min. Ionization by electron impact (70 eV) was carried out at 250 °C with a transfer line temperature of 280 °C. We recorded mass spectral fragments in the *m/z* range between 39 and 550.

The initial tentative identification of the volatile compounds in the fecal samples was carried out by comparing the fragmentation patterns (i.e., mass spectra) of the compounds detected in the samples with those available in the NIST/EPA/NIH 2010 mass spectral library. When possible (83.9% of compounds), the identification was confirmed by comparing the spectra and retention times with those obtained under the same conditions of the analysis using authentic GC grade or high purity standards (from Sigma-Aldrich Chemical Co). Impurities identified in the control vial samples, such as plastics, benzenamines, hydroperoxides, etc., are not reported.

### Statistical analysis

The relative amount of each chemical compound was determined as the percentage of the area of its peak in the chromatogram in relation to the total area occupied by all the peaks (TIC area), excluding contaminants. For this, the integration capacity of the peak areas available in the software Xcalibur (Finningan Co.) was used. For statistical analyses, the relative proportions of each compound were transformed following the formula: log[(proportion)/(1 − proportion)], to correct the problem of non-independence between proportions^58^.

The software PRIMER V6.1.13^59^ and PERMANOVA + V1.0.3^60^ were used to test for differences between the chemical profiles. We calculated the Euclidean distances between every pair of individual samples and produced a resemblance matrix that was the basis of further analyses. We used permutational multivariate variance analyses (PERMANOVA)^61^ based on the Euclidean resemblance matrix, using 999 permutations, to analyse whether the overall chemical profiles of the fecal samples varied between sexes, reproductive status of the individuals (not reproductive vs. reproductive vs. breeding) and in relation to the presumable marking function of feces. Pairwise post-hoc comparisons were made with permutation tests. Differences were further investigated using canonical analyses of principal coordinates (CAP^62^). To determine which compounds differed between categories (sex, reproductive condition, marking function), we used the transformed areas of the compounds that appeared in at least five samples to make a principal component analysis (PCA) with a varimax normalized rotation. The extracted principal components (PCs) were used as new variables to compare categories using one-way analyses of variance (ANOVA). Post-hoc multiple comparisons were made using Tukey's tests^63^. We further used discriminant analyses to test whether a given sample could be assigned to a given category based on the compounds that were significantly correlated with the PC scores that differed significantly between these categories. Statistical analyses were performed using the software Statistica 7.0 (StatSoft Inc., Tulsa, OK).

## Data Availability

The DNA sequences generated in the current study are available in a .fasta file from Dryad repository at: [https://datadryad.org/stash/share/eKtAUkzHwCOV8DabtamcGDRHJpuw4vIK19i4x0-wCRI]. A table listing the faecal samples included in the molecular analyses and their specific and sexual identification is also provided in the same repository.
